# Neuroprotective Effect of Several Phytochemicals and Its Potential Application in the Prevention of Neurodegenerative Diseases

**DOI:** 10.3390/geriatrics1040029

**Published:** 2016-11-12

**Authors:** Jintang Wang, Yuetao Song, Maolong Gao, Xujing Bai, Zheng Chen

**Affiliations:** Institute for Geriatrics and Rehabilitation, Beijing Geriatric Hospital, 118 Wenquan Road, Haidian District, Beijing 100095, China; jtwang6@yahoo.com (J.W.); syt521800@sohu.com (Y.S.); gml3447@163.com (M.G.); 13681096899@126.com (X.B.)

**Keywords:** phytochemical, nursing service, oxidative stress, neuroinflammation, neurodegenerative diseases

## Abstract

The detrimental effects of oxidative stress and chronic neuroinflammation on neuronal cell death have been implicated in the pathogenesis of neurodegenerative disorders such as Alzheimer’s disease (AD) and Parkinson’s disease (PD). The nutritional neuroscience is quickly growing, and phytochemicals or phytobioactive compounds such as curcumin, resveratrol, propolis, ginsenoside, and ω-3 polyunsaturated fatty acids (PUFAs) have been extensively applied to potential therapeutic purposes for numerous neurodegenerative diseases for their anti-oxidative and anti-inflammatory effects. However, their administration as food supplements in the daily diet of the elderly is normally a voluntary and less-organized behavior, indicating the uncertainty of therapeutic effects in this sporadic population; specifically, the effective physiological dosages and the real positive effects in preserving brain health have not yet been fully elucidated. In this review, we collect several lines of evidence on these compounds, which constitute a major type of nutraceuticals and are widely integrated into the daily anti-aging caring of elderly patients, and discuss the underlying anti-oxidative and anti-inflammatory mechanisms of these phytochemicals. In conclusion, we highlight the implications of these compounds in the prevention and treatment of geriatric diseases, and of the potential supplementation procedures used as a dietary therapeutic program in clinical nursing services for patients with neurodegenerative diseases or for the elderly in certain communities, which we hope will lead to more beneficial health outcomes with respect to brain function, innate immunity, and gastrointestinal function, as well as more economic and social benefits.

## 1. Introduction

With the rapid population aging, an advanced age is a major risk factor leading to an increased prevalence of chronic, age-related diseases such as neurodegenerative diseases including Parkinson’s disease (PD), Alzheimer’s disease (AD), and amyotrophic lateral sclerosis (ALS). These diseases especially, as a pervasive health threat to the aged population [1,2,3,4], are a cause of disability and death in the elderly, seriously impacting the physical and mental health of the elderly. Research shows that these health conditions result from the common pathogenesis characterized by progressive neuronal loss and impaired neuronal function as a consequence of chronic inflammation and oxidative stress [5]; therefore, a better understanding of their underlying mechanism will help in the development of strategies for preventing or delaying these disease processes, representing a considerable public health concern and socio-economic burden. In this paper, we focus on the neuroinflammation and oxidative stress, i.e., the common pathologic features or mechanisms [6,7], to address therapeutic approaches linked to the potential administration of dietary nutraceuticals in clinical nursing services or healthcare for the elderly in certain communities.

Substantial evidence shows that a number of dietary or phytobioactive compounds have considerable anti-oxidant and anti-inflammatory effects, displaying an inhibitory role in the oxidative and inflammatory mechanisms associated with neurodegenerative diseases [8,9]. These compounds include polyphenols such as curcumin, resveratrol, ginseng, rosmarinic acid, and other nutritious components such as propolis, ω-3 polyunsaturated fatty acids (PUFAs), and vitamin E, and their anti-oxidative and anti-inflammatory roles have been widely confirmed in vivo and in vitro, including inhibited neurotoxic effects, by eliminating or limiting the activities of the reactive oxygen species (ROS) and reactive nitrogen species (RNS) [10] from the oxidative stress pathway and toll-like receptors, NF-κB and cytokines (TNF-α, IL-6, IL-1β, and IFN-γ), from proinflammatory immune pathways [2,11,12]. Dietary supplementation can improve the recovery and regeneration of dopamine terminals in the striatum in the PD brain, rather than prevent initial damage [13].

Generally, there are at least two interventional strategies available in dietary intake to improve brain health with age. One is caloric restriction, which can control oxidative stress or inflammatory response and promote the release of brain-derived neurotrophic factor (BDNF) to ameliorate brain function [14], and the other is the nutritional intervention by the oral administration of phytobioactive components, antioxidants, or polyphenolic compounds, which can result in decreased pro-inflammatory cytokines and oxidative damage and therefore are a more applicable therapeutic approach with a higher adherence for counteracting neurodegenerative diseases [6,7,15,16]. Meanwhile, it has been demonstrated that the phytobioactive components such as curcumin, propolis, and PUFAs can obviously ameliorate gastrointestinal functions, including gastrointestinal metabolism, motility, and digestive function, and effectively improve constipation, the most common condition among the elderly [17,18].

In this article, we review the potential administration of several types of compounds—polyphenols such as curcumin, resveratrol, propolis, and PUFAs—for preventive and therapeutic purposes based on related research evidence and promising clinical support in the healthcare services of certain hospitals and communities, which involves their anti-aging and anti-inflammatory mechanisms and the possible side effects of a daily diet. Although their nutritional values and therapeutic conception are well-established in society, their availability in daily life for the older population has not been widely accepted, especially low administration adherence, which impacts elderly physical health to varied degrees, a potential risk for the high morbidity and mortality in the older population. Finally, the feasibility of their application procedures in healthcare services for the elderly is discussed, along with the possible improvement of the physical quality of the elderly by enhancing brain function, activating the inherent immune function, potentiating the muscular function, and improving gastrointestinal function. The possible implementation of this dietary therapeutic program will predictably produce profound social and economic benefits for both individual families and government budgets for elderly healthcare.

## 2. Polyphenols and Their Involvement in the Brain Innate Immunity

### 2.1. Resveratrol

Resveratrol is a kind of nonflavonoid polyphenols and mainly exists in berries, peanuts, and medicinal plants, with grapes and red wine being the most substantial source of dietary resveratrol [1,19,20]. Resveratrol has a wide range of biologic properties, including anti-oxidative, anti-inflammatory, and anti-carcinogenic features [1], and neuroprotective effects as evidenced by reduced oxidative stress, namely the reduced production of reactive oxygen species (ROS) and superoxide ions [21,22] in both animal and various cell model studies for neurodegenerative disorders [2]. In clinical trials, the biologic effects of resveratrol were investigated by examining the related variables such as pharmacokinetics, metabolism, safety, tolerance, and bioavailability [20,23,24], which involve several other conditions such as cancer, cardiovascular disease, obesity, and diabetes. Moreover, the relative safety of resveratrol was studied, showing the short-term or acute administration of single or multiple doses (25 mg to 5 g) and minor or inconsistent side effects, but not enough toxicity of chronic intake of resveratrol was described. In addition, in vitro and in vivo studies have shown that resveratrol prevented neurons from β-amyloid (Aβ)-induced toxicity and cell death by the destabilization of Aβ fibrils [25] and exerted an anti-oxidative effect in neurodegeneration and cognitive impairment in the models of sporadic and transgenic AD and tauopathy, along with reduced plaque damages in cortical, striatal, and hypothalamic regions [1,26]. Importantly, the anti-inflammatory and anti-oxidative effects of resveratrol are linked to the suppression of activated NF-κB, sirtuin 1, and MAPK pathways, such as the reduced release of proinflammatory TNF-α, IL-1β, and NO in microglia [26], indicating a potential role in the treatment of neurodegenerative pathology. These studies and human trials demonstrated safety and tolerance, which provide a possibility to promote their administration in the aged population followed by examining neurologic outcome variables in order to establish the therapeutic effects of adjunctive resveratrol in neurodegenerative diseases.

### 2.2. Curcuminoids

Curcuminoids, as the main polyphenol constituents of curcuma longa, consist of three chemical components: curcumin (75%–80%), demethoxycurcumin (15%–20%), and bisdemethoxycurcumin (3%–5%). Multiple lines of evidence show that curcumin is a polyphenolic compound with anti-oxidative and anti-inflammatory properties and is commonly used as a food additive to produce neuroprotective effects by controlling the oxidative and inflammatory mechanisms in the pathogenesis of AD and PD [27,28,29]. The curcumin can effectively suppress inflammatory responses of brain microglia in the models of AD [28,29] and recover Aβ-induced long-term potentiation impairment involving reduced amyloid plaque burden and preformed Aβ fibrils [27], the pathologic hallmark of AD. Furthermore, curcumin can provide protection against α-synuclein-induced cytotoxicity in SH-SY5Y neuroblastoma cells by decreasing cytotoxicity of aggregated α-synuclein, reducing intracellular ROS, inhibiting caspase-3 activation, and ameliorating signs of apoptosis [29]. In addition, the dietary curcumin intake is positively related to cognitive function in healthy elderly individuals, while the consumers of large amounts of curcumin exhibit lower concentrations of Aβ and tau [30]. Nevertheless, the potential therapeutic effects of supplemental curcumin administration still need to be accurately evaluated, because the clinical investigations on the treatment effects of curcumin show indiscernible differences in cognitive scores and the biochemical features of AD patients [31], so a better understanding of its effective dose and bioavailability is needed to develop an appropriate evaluation procedure that involves a much larger sample size, a longer duration, etc. For example, a tolerability of up to 12,000 mg of curcumin was previously reported. Therefore, due to the hydrophobic attribution of curcuminoids, the conjugated curcumins such as nano-curcumin or curcumin-like analogs is to be developed to increase their bioavailability and potential effects for improvement in AD [32,33,34]. Given these numerous beneficial properties, curcuminoids show promise as a therapeutic agent for neurodegenerative diseases.

### 2.3. Other Polyphenols

The quercetin, another form of flavonol polyphenol found in many fruits, vegetables, leaves, and grains, can be used as an ingredient in supplements, beverages, and foods, exhibiting its anti-oxidative and neuroprotective pharmacological properties by engagement in various signaling pathways in a variety of cell and disease models of neurodegenerative disorders. Green tea, rich in polyphenol and its principal constituent epigallocatechin-3-gallate (EGCG), exerts neuroprotective effects by modulating neuroinflammation and ameliorating oxidative stress and neural damage, as evidenced in various studies [2,35].

Taken together, polyphenols are strong antioxidants in vitro and in vivo in both animal models and humans. The habitual consumption of dietary flavonoids is proven to inhibit various secondary sources of reactive oxygen species (ROS) and proinflammatory cytokines and improve mitochondrial bioenergetics, thus reducing the risk of neurodegenerative disorders such as AD, PD, and stroke. A 13-year long clinical study indicated that a higher intake of these antioxidant polyphenols helps improve memory and has the potential to inhibit brain aging [36]. Moreover, these antioxidants have been found to decrease plasma total homocysteine, which contributes to the attenuation of AD pathology [37]. Hence, the clinical translation of polyphenols as an antioxidant therapy is a promising approach to attenuate oxidative damage in aging and age-related disorders, and the combined specific dietary polyphenols selected on the basis of oral bioavailability, brain penetration, and the inhibition of multiple processes responsible for excessive ROS production may be a viable approach for the prevention and treatment of neurodegenerative disorders.

## 3. Propolis and Royal Jelly and Their Involvement in the Brain Innate Immunity

Honeybee propolis is collected from various plants, has been widely used as a folk medicine to maintain health, and has been demonstrated to be neuroprotective on neurodegenerative disorders [38,39]. It has been shown to have a range of biological activities such as anticancer, anti-inflammatory, antibiotic, ant-oxidative, antifungal, anesthetic, and cytostatic effects, which are principally attributed to the presence of flavonoids (the major component plus rutin, quercetin, galangin, etc.), phenolic compounds, and caffeic acid phenyl ester (CAPE) [38,39]. Recent studies have confirmed that propolis supplementation can reduce kainic acid-mediated exicitotoxicity in the brain by the inhibitory effects on oxidative stress and activated proinflammatory cytokines, suggesting that propolis can protect against neuronal damage [38,40]. Meanwhile, the concentrations of TNF-α and NO, along with the activities of NOS and caspase-3 in the brains of rats, can be used as effective evaluation indicators for the neuroprotective effect of propolis [38], indicating a tight correlation of the beneficial effects of propolis with its anti-oxidative, anti-inflammatory, and anti-apoptotic roles. In addition, our primary clinical trial indicates that the propolis and royal Jelly have a curative effect on gastrointestinal dysfunction [17], constipation [18], and muscular dysfunction [41], all of which are the most common disorders associated with the elderly; thus, it is called “liquid gold”, showing its therapeutic significance in healthcare and clinical services for the aged population.

Caffeic acid phenethyl ester (CAPE) is an active component of propolis and a resinous mixture obtained from honeybee hives, which has been demonstrated to exhibit anti-inflammatory and anti-oxidative properties [42]. For example, CAPE can inhibit NF-κB signaling and protect neurons from a number of insults such as inflammatory stress and oxidative stress [43,44]. The CAPE and phenolic compounds in propolis have been shown to penetrate cellular membranes and cross the blood–brain barrier [45,46,47] and protectively control the loss of dopaminergic neurons induced by 6-OHDA in rats [48], involving the amelioration of oxidative stress or NO concentration, and the reduced oxidative stress markers and expression of pro-inflammatory cytokines [40]. In addition, CAPE has been shown to induce heme oxygenase-1 (HO-1) expression in several types of cells in vitro [48], indicating its potential protective effect on dopaminergic neurons. In the organotypic midbrain slice cultures in vitro and lipopolysaccharides (LPS)- or 6-hydroxydopamine (6-OHDA)-injected mouse models of PD in vivo, a higher concentration (30 mM) of CAPE suppressed NO production, as evidenced by the inhibited NF-κB activation and iNOS gene expression [49], leading to the protection of dopaminergic neurons. It has been demonstrated that CAPE exhibits a novel feature as an inducer of BDNF expression in addition to its known ability to induce HO-1 expression, and both HO-1 and BDNF may contribute to the neuroprotective effect of CAPE.

## 4. PUFAs and Their Involvement in the Brain Innate Immunity

The PUFAs are classified into two biologically important families: ω-3 fatty acids, such as alpha linolenic acid (ALA), docosahexaenoic acid (DHA), and eicosapentaenoic acid (EPA), and ω-6 fatty acids, such as linoleic acid and arachidonic acid (AA) [50]. DHA and AA are abundant in the brain and are highly susceptible to free radical attack in brain aging and disorders [51,52], so the habitual supplementation of dietary PUFAs is an effective approach to counteract age-related neurodegenerative diseases or cardiovascular diseases and thus to potentially mitigate the morbidity and mortality of the elderly suffering from these diseases. Oxidative damage of the brain is characterized by increased lipid peroxidation, while the redox changes in membrane fatty acid composition have been shown to contribute to the deterioration of neuronal functions, as a consequence of dysregulated lipid metabolism and signaling in several neurodegenerative diseases [53]. Research shows that ω-3 PUFAs are an important regulator of many metabolic and inflammatory pathways and participate in pleiotropic pathological activities in the brain. DHA especially is found in high concentrations in the brain (about 40% of neural phospholipids in plasma membrane), showing its intrinsic neuroprotective implications [54,55]. DHA is shown to decrease with cognitive decline in both healthy aged adults [55] and AD patients, as evidenced by postmortem samples from AD brains [56,57]. On the other hand, the higher dietary intake of DHA and the higher concentrations of plasma DHA can reduce the associated risk of cognitive impairment or AD [57,58]. As such, clinical investigations in multiple profiles have been performed to evaluate the therapeutic efficacy of ω-3 PUFAs in age-related disorders [1,59]. ω-3 PUFAs can enhance learning and memory function in age-related cognitive decline in healthy elderly populations, but cannot benefit patients with already diagnosed AD progression [60,61], which may be helpful and promising in the design of their applications in the future.

Recently, the conception of introducing DHA as an adjunctive therapy for neurodegenerative diseases has been further reinforced based on its molecular action mechanism. DHA deficiency can activate caspases in AD models and exacerbate the age-related decline of glutamatergic transmission in learning and memory functions in rats [62,63]. By contrast, DHA supplementation has been demonstrated to diminish oxidative stress or lipid peroxidation and protect against memory loss in models of AD and aging rats [64] and to reduce the accumulation of neuronal Aβ and tau protein [65], the hallmark of AD pathology. On the other hand, as integral membrane lipids contribute to the maintenance of the structure and function of cell membranes, PUFAs (mainly DHA and AA) can be incorporated into neuronal membranes and reduce the total cholesterol fraction, leading to increased membrane fluidity that is essential to maintain synaptic structures and improve neurotransmission [66,67]. Meanwhile, PUFAs can be converted into essential membrane phospholipids and second messengers to modulate inflammatory response, oxidative stress, and neuronal function [68]. Moreover, a high dietary intake of ω-3 PUFAs has been shown to increase gray matter volume in cortico-limbic circuitry, which represents the effective input for memory formation and cortical arousal in the brain [69]. Another kind of study is the investigation of the therapeutic efficacy of ω-3 PUFAs (i.e., DHA and EPA) found in fish oil as anti-inflammatory, antioxidant, and neuroprotective agents [51,70]. It has been demonstrated that fish oil-treated patients with multiple sclerosis (MS) display a significant reduction in the levels of proinflammatory cytokines and NO catabolites, but no variation in the serum levels of lipoperoxides or the number of recurrences per year [54], indicating that the use of antioxidants, even in combination with conventional immunomodulatory therapies, could have synergistic effects on disease progression, leading to more powerful therapeutic outcomes.

## 5. Ginsenosides and Their Involvement in the Brain Innate Immunity

Ginsenosides, the major pharmacologically active ingredients of ginseng, have a wide range of therapeutic and nutraceutical implications and can produce diverse curative effects. They often called “cure-alls” for their anti-inflammatory, antioxidant, anti-aging, immunomodulatory, anticancer, and anti-fatigue effects in rodents and humans [71,72,73]. Thus, ginseng, a herbal medicine, is a natural remedy that can improve immune function by enhancing phagocytosis, natural killer cell activity, and the production of interferons and can promote a resistance to various stresses involving psychological function, cardiac function, and exercise performance [74]. The bioactive components in the ginseng root include approximately 60 ginsenosides in two main structural classes: the 20 (*S*)-protopanaxadiol (PD) group of Ra1-3, Rb1-3, Rc, Rd, Rg3, and Rh2 and the 20 (*S*)-protopanaxatriol (PT) group of Re, Rf, Rg1-2, and Rh1 [75]. Currently, researchers mainly focus on the purified individual ginsenosides to reveal the specific mechanism of ginseng functions instead of all ginseng root extracts, showing that ginsenosides may have different effects in pharmacological mechanisms due to their different chemical structures, with the most commonly studied ginsenosides being Rb1, Rg1, Rg3, Re, Rd, and Rh1.

Mounting evidence in vitro and in vivo shows that ginsenosides have anti-oxidative and anti-inflammatory properties and neuroprotective effects in their application to neurodegenerative disorders such as AD and PD [75,76,77,78,79]. These pharmacological mechanisms acting on brain disorders involve the attenuation of excitotoxicity, oxidative stress and neuroinflammation, the maintenance of neurotransmitter balance, anti-apoptotic effects, and mitochondrial stabilization effects [80,81,82], by which ginsenosides regulate the underlying pathological mechanisms and improve the cognitive function and brain activities in several neurodegenerative diseases. For example, Rg1 is a potential regulator of cytokines such as TNF-α and IL-1β that reduces α-synuclein-mediated neuroinflammation [83], and a mediator of hypoxia-inducible factor-1a (HIF-1a) that acts via this transcription factor to improve cell survival, angiogenesis, and neurogenesis [72,84]. Oral treatment with pseudoginsenoside-F11 (PF11), a component of Panax quinque folium, can increase the activity of anti-oxidant enzymes superoxide dismutase (SOD) and glutathione peroxidase (GSH-Px), and improve the ability of learning and memory in a mouse model of AD [85]. In addition, Rb1, Rg1, Rd, and Re have been shown to be neuroprotective agents for PD [75,77,82,83] due to the inhibition of oxidative stress and neuroinflammation and the decrease in toxin-induced apoptosis and nigral iron levels [86]. Oral administration of ginseng extract G115 can modulate dopaminergic neuronal loss in the substantia nigra and reduce locomotor dysfunction in the 1-methyl-4-phenyl-1,2,3,6-tetrahydropyridine (MPTP) and 1-methyl-4-phenylpyridinium (MPPC) models of PD [83,87].

Animal and cell culture studies have indicated that ginsenosides can reduce free radical production and enhance brain function, showing different activities in both physiological and pathologic conditions, but their action mechanisms, specificity, structure and function relationship, detailed pharmacokinetics, and toxicity have not yet been fully elucidated in both animal models and humans. Thus, further development of ginseng compounds as effective therapeutic agents is still required. AD and PD are typical neurodegenerative disorders for which most evidence for the benefits of ginseng has been obtained, pre-clinically and through a small number of clinical studies, but the therapeutic efficacy of ginseng and ginsenosides in the prevention and treatment of these disorders, and possibly other neurological conditions, needs to be further confirmed. It is hoped that the data offered here can bring about a better understanding of ginseng pharmacologic properties and stimulate more studies on dietary application procedures to potentiate the beneficial effects of ginseng on patients with neurodegenerative disorders and on healthy older residents in certain communities.

## 6. Conclusions and Perspectives

Nature has packaged a wide array of phytochemicals that likely act in synergy to promote health and prevent aging. Currently, the nutritional neuroscience is quickly growing, and the therapeutic effects of whole foods and herbs as neuroprotective agents have become the focus of research by neuroscientists [88,89], especially the health-beneficial aspects of phytobioactive components involving the reduction of oxidative stress and of immune-mediated inflammation and the exposure of various environmental factors and behavioral determinants, such as diet and exercise [90]. In fact, in the habitual diet of the elderly, the phytochemicals discussed in this review have been used to produce potential therapeutic values for numerous neurodegenerative diseases, but the rate of regular administration in the aged population is limited and needs to be potentiated through the education and popularization of relevant nutritional procedures.

Collectively, the nutritional intervention can produce three major positive outcomes in the prevention and treatment of the geriatric diseases: the neuroprotective and anti-oxidative effect [22], the immunity-enhancing and anti-inflammatory effect, and the gastrointestinal improvement and nutritional effect. Therefore, as a well-established conception, the regular administration of these phytochemicals is an important approach to enhance the basic nutrition of the elderly, a prerequisite for the maintenance of physical health, the reversal of brain function decline and disease resistance capability. It is strongly suggested that their routine application is performed in preclinical care and treatment of patients with AD and PD, or in the healthcare of the elderly at home or nursing institutions. While their daily administration is considered at the appropriate dosages and reasonable compatibility with other medicines, their toxic and side effects are generally lacking and not taken into account based on their pharmacological properties. Although this approach often results in a rigorous lifestyle change, this will still have a better therapeutic adherence than the caloric restriction approach, and will be easily accepted as nutraceuticals [91]. Moreover, the synthetic bioactive compounds have various toxicity limitations and are still extensively used due to their anti-oxidative or anti-inflammatory attribution. Among the phytochemicals, the phenolic natural compounds (phenolic acids and flavonoids) are the most beneficial [34], and oral administration is the most convenient for the repeated and routine delivery of these compounds, but the most challenging issue is how to cross the blood–brain barrier to protect the brain.

In summary, phytochemicals exhibit a remarkable multipotent ability to control and modulate oxidative stress, chronic inflammation, and mitochondrial dysfunctions, three hallmarks of neurodegeneration [2]. To reduce the prevalence of neurodegenerative diseases and their pervasive health threat to the growing aged population, it is necessary to establish novel preventive and intervention procedures available in clinical nursing services for patients or healthy adults by direct usage and dietary supplementation of phytochemicals. The lack of toxic effects and the easy acquisition from natural sources are advantageous for adoption and generalization of dietary therapeutic programs in the aged population. Future research needs to aim towards a clinical acceptance of health claims from preclinical studies in vitro and in vivo, and human clinical trials of several potent compounds and their combinations should be carried out, including risk assessments and safety evaluations to observe any undesirable effects. The success in clinical research of polyphenols will decide their pharmacological relevance for humans, and nutritional intervention programs may decrease oxidative damage, slow the rate of aging, lessen the risk of neurodegenerative disorders, and increase the lifespan of older adults to achieve the goal of healthy aging [92], so that older adults can remain both physically and cognitively healthy into older age, with reduced social and economic burdens.
